# In situ magnesiothermic reduction synthesis of a Ge@C composite for high-performance lithium-ion batterie anodes

**DOI:** 10.3762/bjnano.14.62

**Published:** 2023-06-26

**Authors:** Ha Tran Huu, Ngoc Phi Nguyen, Vuong Hoang Ngo, Huy Hoang Luc, Minh Kha Le, Minh Thu Nguyen, My Loan Phung Le, Hye Rim Kim, In Young Kim, Sung Jin Kim, Van Man Tran, Vien Vo

**Affiliations:** 1 Faculty of Natural Science, Quy Nhon University, 170 An Duong Vuong, Quy Nhon, Binh Dinh, 55000, Vietnam; 2 Faculty of Physics, Hanoi National University of Education, 136 Xuan Thuy, Cau Giay, 11300, Hanoi, Vietnam; 3 Applied Physical Chemistry Laboratory, University of Science, Viet Nam National University Ho Chi Minh City, 70000, Vietnam; 4 Department of Chemistry and Nanoscience, Ewha Womans University, Seoul 120-750, South Korea

**Keywords:** Ge anode, in situ synthesis, lithium-ion batteries, magnesiothermic reduction

## Abstract

Metallothermic, especially magnesiothermic, solid-state reactions have been widely applied to synthesize various materials. However, further investigations regarding the use of this method for composite syntheses are needed because of the high reactivity of magnesium. Herein, we report an in situ magnesiothermic reduction to synthesize a composite of Ge@C as an anode material for lithium-ion batteries. The obtained electrode delivered a specific capacity of 454.2 mAh·g^−1^ after 200 cycles at a specific current of 1000 mA·g^−1^. The stable electrochemical performance and good rate performance of the electrode (432.3 mAh·g^−1^ at a specific current of 5000 mA·g^−1^) are attributed to the enhancement in distribution and chemical contact between Ge nanoparticles and the biomass-based carbon matrix. A comparison with other synthesis routes has been conducted to demonstrate the effectiveness of contact formation during in situ synthesis.

## Introduction

The significantly increasing energy consumption leads to the exhaustion of fossil fuel sources such as coal, oil, and natural gas. Additionally, there are various negative environmental consequences of using fossil fuel energy, such as water pollution, increasing emissions of greenhouse gases, and air pollution [1]. Therefore, research regarding eco-friendly and renewable energy resources has emerged [2]. One of the best alternatives to fossil fuels are solar and wind energy [3]. However, high-power, high-energy, and long-lasting energy storage systems are necessary to utilize these energy resources effectively [4]. Moreover, to reduce greenhouse gas emissions, various governments have committed themselves to develop strategies for increasing the number of electric vehicles (EVs) [5–6]. The most important component of EVs are suitable energy storage systems, the further development of which will be key to a more widespread use of this kind of transportation [7].

Commercialized first by Sony company, lithium-ion batteries (LIBs) and related systems have become the most popular energy storage systems, with applications from mobile devices to EVs and grid-scale storage [8–9]. However, the low specific theoretical capacity of graphite limits the energy density of the commercial LIBs [10–13]. Germanium, as a lithium alloying material, is a possible alternative for graphite electrodes due to its high theoretical capacity of 1623 mAh·g^−1^ (four times higher than that of graphite) and good rate performance due to high electronic (2.1 S·m^−1^, 1 × 10^4^ times higher than that of silicon) and ionic (6.51 × 10^−12^ cm^2^·s^−1^, 400 times higher than Si at room temperature) conductivity [14]. However, electrodes based on alloying suffer from deterioration during repeated lithiation/delithiation because of the large variation in volume. Despite the lower volume change ratio compared to Si (370% for Ge and 400% for Si) and the structural robustness, for example, a higher resistance to pulverization than Si, the durability of Ge-based anode is not sufficient for practical applications [14]. To overcome this limitation, nanoscale control and composite design are two effective strategies [15–19].

In addition to various Ge preparation routes, such as sputtering deposition [20], wet-chemical reduction [21–22], thermal reduction [23], colloidal synthesis [24], and molten-salt synthesis [25], metallothermic, especially magnesiothermic reduction, has been widely applied in the synthesis of group-IV elements to control the nanostructures of the obtained products because of its simplicity in operation and the applicability for pseudomorphic transformations [26–28]. For instance, in our previous study, a magnesiothermic reaction was applied for the reduction of GeO_2_ to Ge nanoparticles [29]. In addition to improving the cycling performance of Ge-based anodes, a carbon matrix is the most popular choice to disperse nanoparticles, avoiding their aggregation and reducing the internal stress induced by volume variation, because of its flexible structure and high conductivity [30–32]. In our recent study, the combination of Ge nanoparticles and a carbon matrix using a hydrothermal route has been reported, and the enhancement in the electrochemical performance of Ge@C electrodes was demonstrated [33]. In this work, a one-pot synthesis route has been followed to prepare a Ge@C composite using an in situ magnesiothermic reduction of GeO_2_ and biomass-derived carbon as precursor. A series of experiments using other methods to combine Ge and biomass carbon was also conducted for comparison. The in situ synthesized electrode exhibits superior electrochemical performance in lithium storage. This is attributed to a better contact between the components obtained via this route.

## Results and Discussion

### Physicochemical characterization

**Figure 1:**
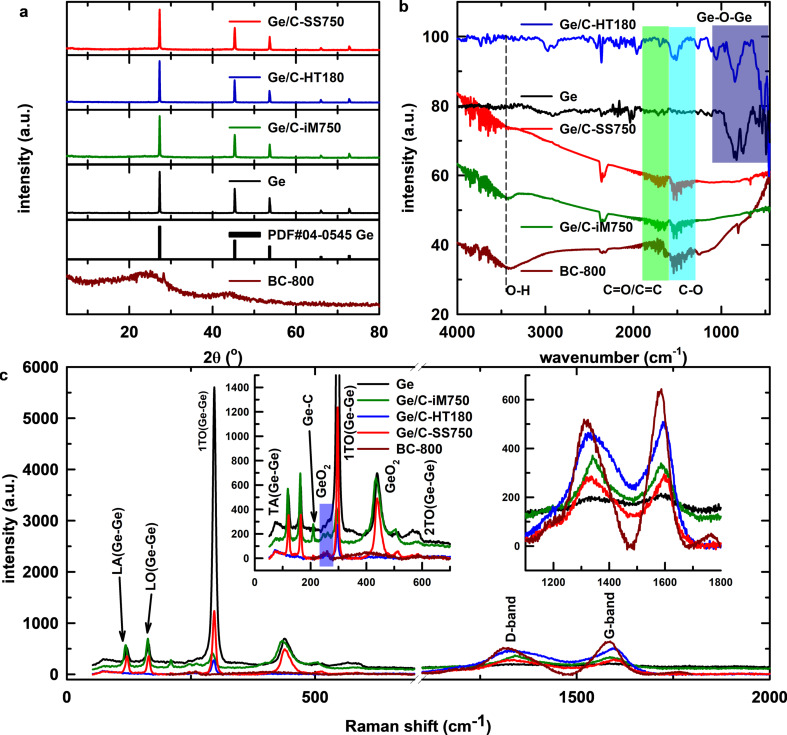
(a) XRD patterns, (b) FTIR spectra, and (c) Raman spectra of pure Ge, Ge/C-iM750, Ge/C-HT180, Ge/C-SS750, and BC-800 (see Experimental section for sample denominations).

The synthesis of Ge/C-iM750 (see Experimental section for sample denominations) was carried out through a one-pot in situ generation of Ge nanoparticles in the presence of a carbon matrix. In this synthesis route, the newly formed nanoparticles are deposited uniformly inside the carbon matrix, which is favorable for the formation of good chemical contacts between the components. The magnesiothermic reduction of GeO_2_ to form Ge is given in Equation 1. Accordingly, the stoichiometric molar ratio of Mg and GeO_2_ should be 2:1. However, in the previous report [29], up to a molar ratio of 2.5, the GeO_2_ phase still remained. Therefore, in this work, Mg and GeO_2_ powders were mixed at a mass ratio of 5:4 (approximately a molar ratio of 3.5:1) to ascertain the formation of the pure Ge phase. The XRD patterns of pure Ge, biomass-derived activated carbon, and as-synthesized Ge@C composites are shown in Figure 1a. The pattern of Ge pattern exhibits reflections at 2θ = 27.3°, 45.3°, 53.7°, 66.0°, and 72.8°, corresponding to the (111), (220), (311), (400), and (331) crystal planes of cubic Ge (space group F*d*−3*m*, JCPDS card No. 04-0545). There is no observable signal related to the GeO_2_ precursor. The XRD pattern of the BC-800 carbon material exhibits a diffraction signal at 2θ = 26.3° attributed to the (002) plane of disordered graphite-like carbon. The peaks at 2θ = 28.1° and 44.0° correspond to the (104) and (100) planes of the hexagonal structure in graphite [34–35]. However, the intensity of the diffraction peaks in the XRD pattern of BC-800 is mostly weak, which indicates the poor graphitic structure with small crystallite sizes of biomass carbon [36]. In the Ge@C composite sample, signals related to the Ge phase are observable, while those of the carbon phase do not appear. There are no significant differences between the XRD patterns of pure Ge and the composites, which implies that the presence of carbon does not alter the structure of Ge.


[1] $$2\text{Mg}+{\text{GeO}}_{2}\overset{\Delta }{\to }2\text{MgO}+\text{Ge}$$

Chemical bonds and the carbon phases in the composites were determined using FTIR spectroscopy, as shown in Figure 1b. The FTIR spectra of pure Ge consist of two broad bands in the wavenumber ranges of 500–600 and 800–1000 cm^−1^. The former band corresponds to the bending modes, and the latter band is attributed to the stretching modes of Ge–O–Ge bonds [37] from the oxidized outer layer of the Ge nanoparticles. The FTIR spectrum of BC-800 exhibits absorption bands at ca. 3500, 1400–1600, and 1000–1300 cm^−1^. The band in the high-wavenumber region is ascribed to the vibration of hydroxy O–H bonds [38]. The medium-wavenumber signals are assigned to the stretching motions of C=O and C=C groups. The remaining band are stretching vibrations of alkoxy C–O or aromatic bonds [39].

Further analysis of the structure of pure Ge, biomass-derived carbon, and chemical contact between Ge and carbon matrix in the composites was conducted using Raman spectra. As shown in Figure 1c, the Raman spectrum of pure Ge exhibits a signal at 83 cm^−1^, attributed to the transverse acoustic phonon mode, and two peaks at 127 and 169 cm^−1^ ascribed, respectively, to the longitudinal acoustic and longitudinal optic phonon modes of Ge–Ge bonds [40–41]. The sharp peak at 296 cm^−1^ and a low-intensity band around 550 cm^−1^ are assigned to the first-order and second-order transverse optic phonon modes of crystalline Ge [41–43]. Moreover, the broadband detected at 449 cm^−1^ corresponds to the customary Raman-active motion of α-GeO_2_ [44]. The presence of characteristic signals in the Ge@C composite is similar to that of the samples prepared through other direct coupling methods (Ge/C-HT180 and Ge/C-SS750), except for the emergence of two bands at 1354 and 1608 cm^−1^, D band and G band, respectively, which signify disordered and graphitic phases of carbon-based materials [45–46]. The difference in Raman signals between Ge/C-iM750 and pure Ge is more notable. New peaks appear at 252, 264, 438 and 509 cm^−1^, indicating further in situ surface oxidation of newly formed Ge and functional groups containing oxygen on the carbon material [47].

The intensity ratio of the Ge–Ge (ca. 300 cm^−1^) and Ge–O (ca. 440 cm^−1^) signals can be used to assess the thickness of the oxide layer [48]. The *I*_Ge–O_/*I*_Ge–Ge_ ratio shows a significant increase from 0.134 (pure Ge) to 1.68 (Ge/C-iM750). The ratio of Ge/C-SS750 is *I*_Ge–O_/*I*_Ge–Ge_ = 0.721, which may be explained by the fact that oxygen-containing functional groups of biomass carbon induce the oxidation of Ge [47]. However, the lower ratio observed in Ge/C-SS750 suggests a less tight contact between Ge and carbon matrix than in Ge/C-iM750. In addition, the emergence of a new signal at 208 cm^−1^ and the decreased intensity of the transverse optic mode of crystalline Ge suggest the development of a novel Ge–C bond, akin to what has been observed in earlier studies on Ge–Sn and Ge–graphene [49–50]. Upon closer inspection of the Raman spectra between 1200 and 1800 cm^−1^, it can be observed that the *I*_D_/*I*_G_ intensity ratio increases from 0.79 for BC-800 to 1.07 for Ge/C-iM750. This indicates that the graphite structure has undergone deformation, resulting in a highly disordered carbon matrix. Such a transformation is anticipated to enhance the conductivity and to increase the number of active sites for binding lithium ions [51–52]. These results demonstrate the enhanced contact between the in situ formed Ge and the carbon matrix, which promotes conductivity, accommodates volume variation, and ultimately improves the electrochemical performance of the material.

**Figure 2:**
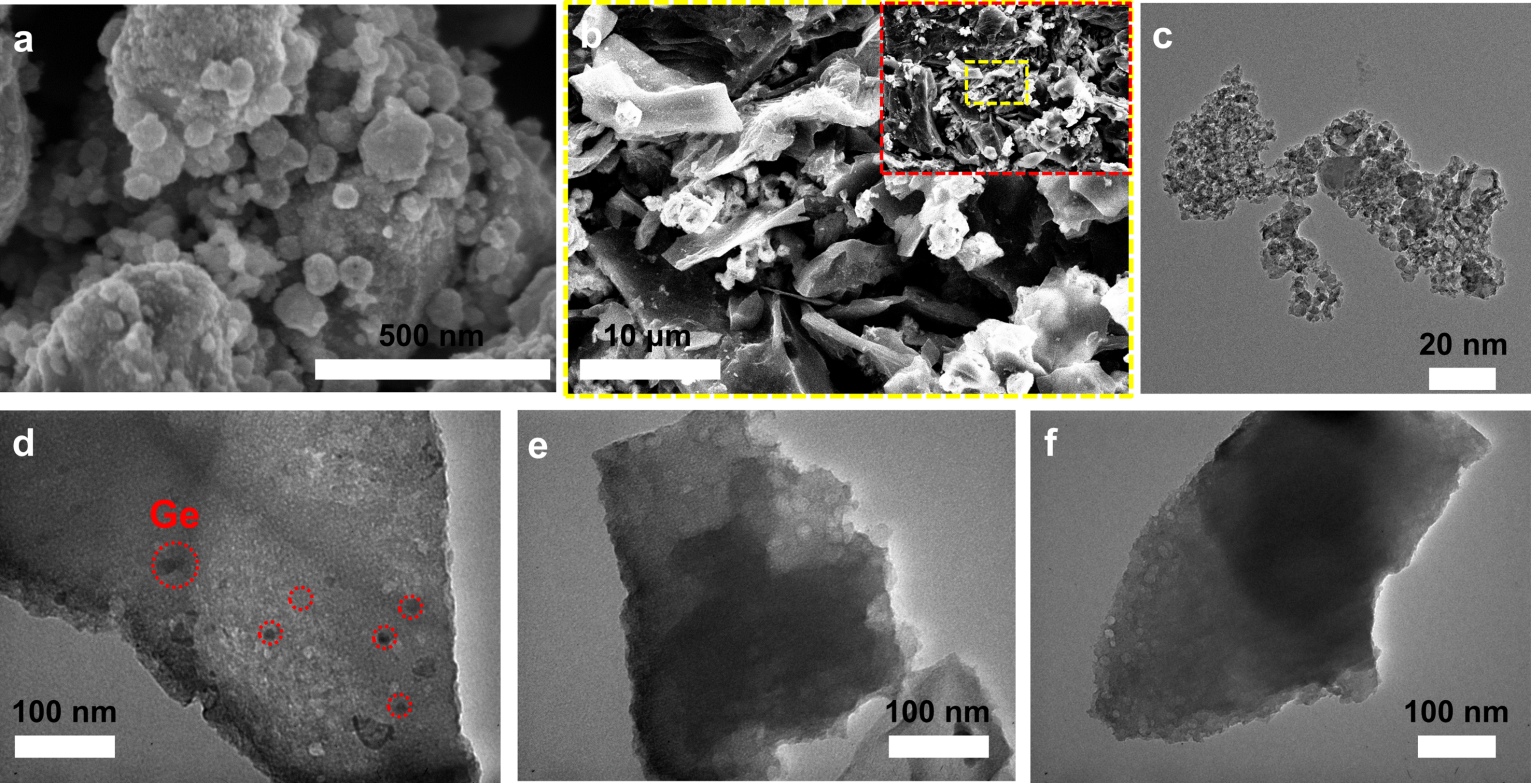
SEM images of (a) Ge and (b) Ge/C-iM750; TEM images of (c) Ge, (d) Ge/C-iM750, (e) Ge/C-HT180, and (f) Ge/C-SS750.

The morphology of the materials was observed using FE-SEM and HR-TEM. As shown in Figure 2a, the spherical Ge nanoparticles are quite uniform, with dimensions of several nanometers. In Figure 2b, the composite Ge/C-iM750 combines Ge nanoparticles with porous activated carbon. The texture of Ge@C composites was observed using TEM. According to Figure 2d–f, the Ge nanoparticles in Ge/C-iM750 are well dispersed in the porous carbon matrix, while they seem to be aggregated on the carbon surface in Ge/C-HT180 or unevenly distributed in Ge/C-SS750.

### Electrochemical characterization

**Figure 3:**
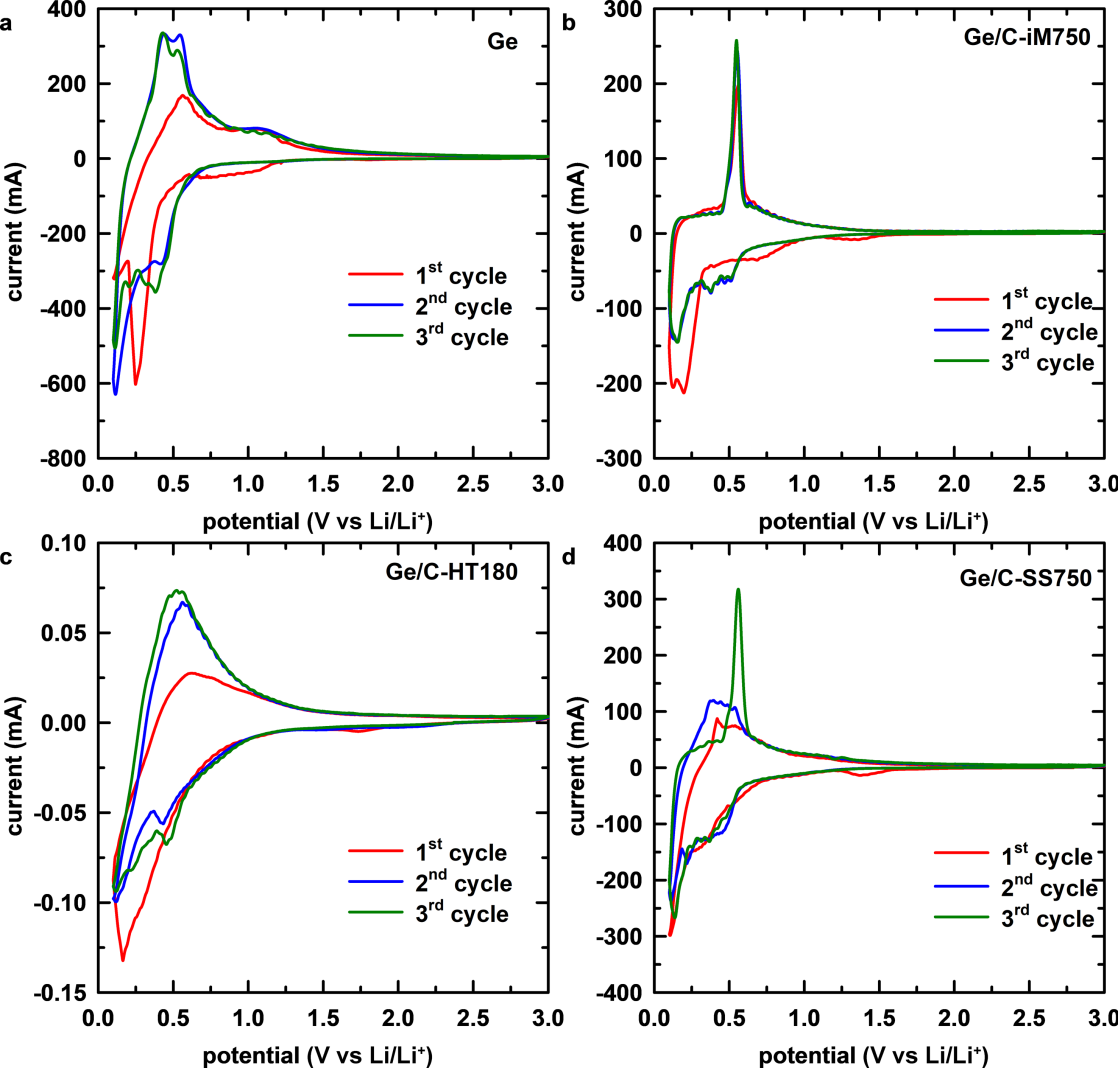
CV measurements of (a) pure Ge, (b) Ge/C-iM750, (c) Ge/C-HT180, and (d) Ge/C-SS750 electrodes.

The electrochemical behavior during lithiation/delithiation of Ge and Ge@C electrodes was investigated using CV curves. As shown in Figure 3a, the first cathodic process of the Ge electrode yielded a reduction peak at 0.17 V vs Li/Li^+^ and a broad band from 1.0 to 0.6 V vs Li/Li^+^. The lower-potential peak with stronger intensity can be attributed to the lithiation of Ge to form Li*_x_*Ge alloys with various compositions such as Li_7_Ge_2_, Li_9_Ge_4_, and Li_22_Ge_2_ [53–55]. The remaining shoulder can be ascribed to the decomposition of the electrolyte and the formation of solid–electrolyte interface (SEI) layers [55–56]. In the following cycles, the signal of the SEI layer formation at a potentials of 0.3 V vs Li/Li^+^ remains, demonstrating the continuous loss of lithium in this irreversible process. This is consistent with the fact that the alloying electrodes suffer from damages caused by the volume variation during lithiation/delithiation, leading to the continuous exposure of new active material and electrolyte [57–58]. In the first anodic process, the peaks at 0.30 and 0.56 V vs Li/Li^+^ correspond to the dealloying reaction of Li*_x_*Ge alloys. This multistep delithiation correlates to the stepwise alloying observed by the splitting of cathodic peaks in the following cycles. Compared to the signals in the first cycle, the subsequent cycles yield broad and low-intensity peaks caused by the electrochemical milling of the active material, leading to reduced size and amorphous structures [59–60].

For the Ge@C electrodes, the first cathodic peaks in the potential range of 0.5–1.5 V vs Li/Li^+^, are assigned to the formation of the SEI layer. They mostly disappear in the subsequent cycles, which demonstrates the buffering role of the carbon matrix in accommodating volume variation and stabilizing the SEI layer [61]. Besides the peak at 0.2 V vs Li/Li^+^ in the first cycle, corresponding to the alloying of lithium ions, a new signal was observed at 0.1 V vs Li/Li^+^, ascribed to the intercalation of the carbon structure [55]. In the following cycles, multistep alloying processes of Ge appear in the range of 0.1–0.8 V vs Li/Li^+^. In the anodic process, the deintercalation of lithium ions from carbon (0.1 V vs Li/Li^+^) and from Li*_x_*Ge alloys (0.3–0.6 V vs Li/Li^+^) is similar in all three Ge@C electrodes. However, the CV curve of Ge/C-iM750 exhibits the highest repeatability. This demonstrates the high stability and reversibility during lithiation/delithiation of this electrode, which could be explained by the better chemical contact between two components, as previously discussed. Noticeably, compared to the Ge component, the reversibly electrochemical signals of carbon (0.1 V for carbon and 0.56 V for Ge) are much lower. This partly indicates that the contribution of the carbon matrix to the total capacity of the electrode is negligible.

**Figure 4:**
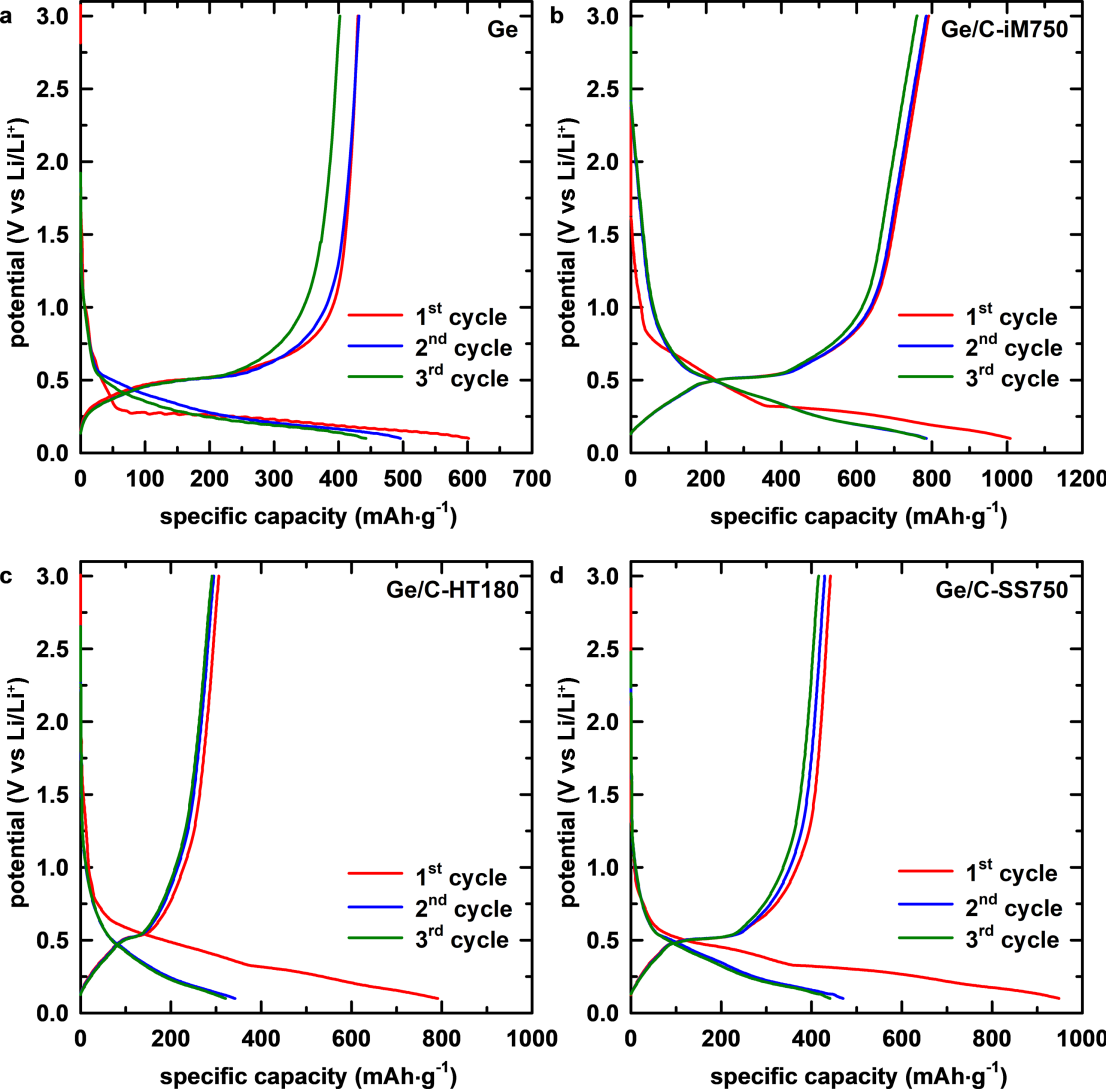
GCPL profiles for the three first cycles of (a) pure Ge, (b) Ge/C-iM750, (c) Ge/C-HT180, and (d) Ge/C-SS750 electrodes.

The galvanostatic cycling with potential limitation (GCPL) profiles of all electrodes in the first three cycles are shown in Figure 4. The first discharge curve of the Ge electrode consists of two plateaus at 0.20 and 0.40 V vs Li/Li^+^, corresponding to the alloying of Li and Ge and the formation of the SEI layer. In the first charge process, the plateau at 0.52 V vs Li/Li^+^ is attributed to the dealloying of Li*_x_*Ge. The long plateau from 0.65 to 0.25 V vs Li/Li^+^ illustrates the continuous irreversible SEI formation in the subsequent cycles. Meanwhile, the signal of SEI formation (plateau at 0.75 V vs Li/Li^+^) in the first cycle vanished in the next discharge, consistent with the CV observations. Compared to the pure Ge electrode, the Ge@C electrodes exhibit lower specific discharge capacity and Coulombic efficiency (CE) values in the first cycle. As summarized in Table 1, the pure Ge electrode delivers specific discharge/charge capacities of 601.7/430.1 mAh·g^−1^, corresponding to a value of the first CE of 71.5%, while the values of Ge/C-HT180 and Ge/C-SS750 are 795.6/307.7 and 946.7/441.2 mAh·g^−1^, with values of the first CE of 38.7% and 46.6%, respectively. The increase in the first specific discharge capacities and the decrease in the initial CE values of Ge@C electrodes can be attributed to the large contribution of the irreversible SEI formation because of the large surface area of biomass-derived activated carbon [62–64], which is electrochemically inactive, as observed in the CV results.

**Table 1:** Summarized results of the electrochemical performance of all electrodes (the specific current was 100 mA·g^−1^ for the first five cycles and 1000 mA·g^−1^ for the subsequent cycles).

Electrode	First specific discharge capacity (mAh·g^−1^)	First Coulombic efficiency (%)	Specific discharge capacity at the 200th cycle (mAh·g^−1^)	Retention at 200th cycle (%)

Ge	601.7	71.5	74.8	41.7
Ge/C-iM750	1008.5	78.5	454.2	95.2
Ge/C-HT180	795.6	38.8	168.3	86.2
Ge/C-SS750	946.7	46.6	204.8	90.2

The first discharge/charge capacities of the Ge/C-iM-750 electrode are 1008.5/791.7 mAh·g^−1^, accounting for value of the first CE of 78.5%. The improved initial CE of Ge/C-iM750 could be attributed to the effect of the in situ magnesiothermic reduction on the carbon structure, as observed in Raman results, as well as the enhanced chemical contact between Ge and carbon matrix. In the subsequent cycles, the CE of all samples increases and reaches approximately 100%, demonstrating the good reversibility of these electrodes. Nevertheless, the slower increase in CE of Ge in the first few cycles is indicative of the unstable SEI of this electrode.

**Figure 5:**
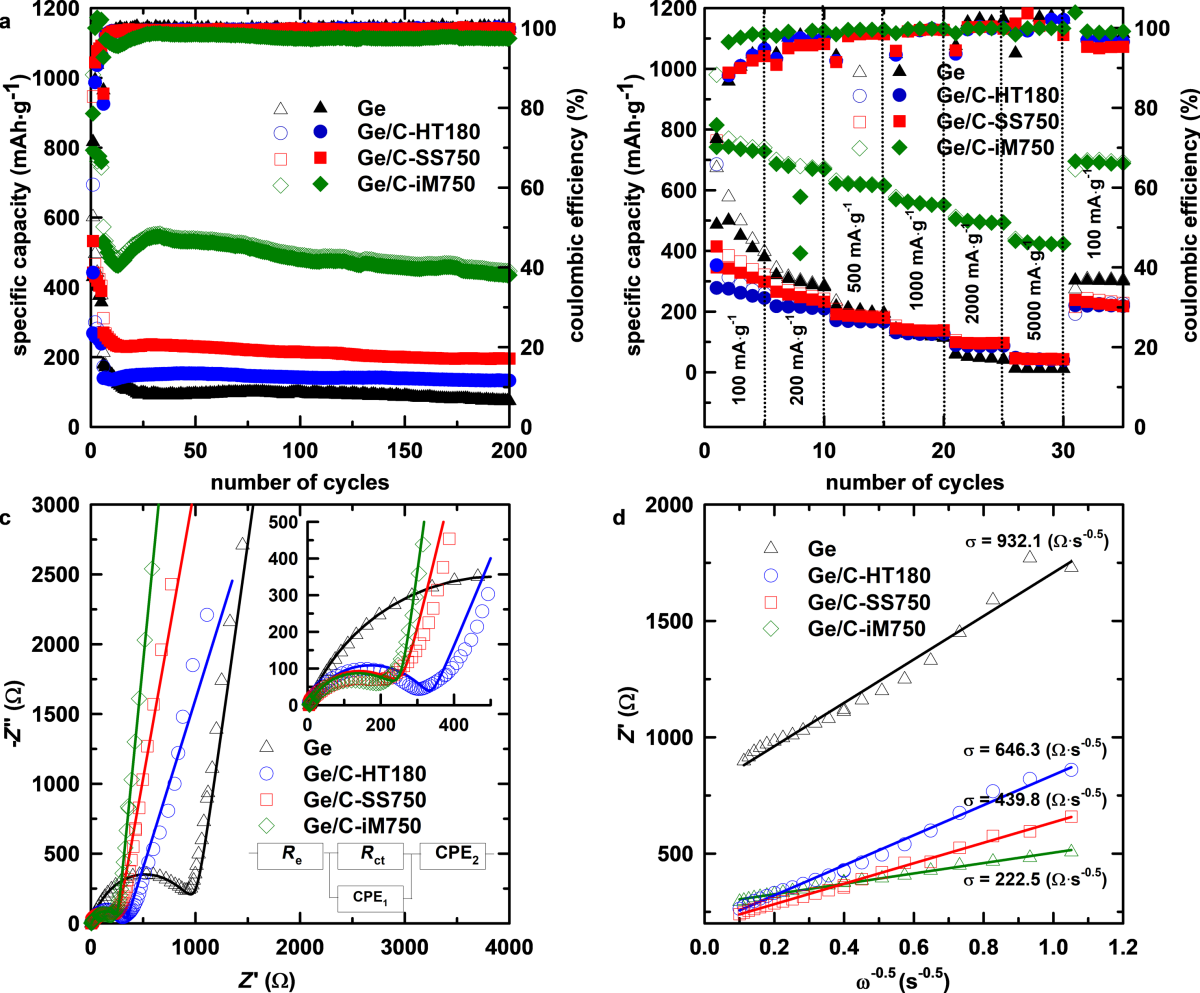
(a) Cycling, (b) rate performance, (c) Nyquist plots of pure Ge, Ge/C-iM750, Ge/C-HT180, and Ge/C-SS750 electrodes, and (d) fit results of the real part of the impedance (*Z*′, Ω) as a function of the angular frequency to the power of −0.5 (ω^−0.5^, s^−0.5^).

The specific capacity values as function of the number of cycles are shown in Figure 5a. After increasing the specific current to 1000 mA·g^−1^, the Ge electrode exhibits a rapid capacity fading to 74.8 mAh·g^−1^, with a retention of 41.7% (compared to the sixth cycle) after 200 cycles. This is explained by the breakdown of Ge particles during continuous lithiation/delithiation, which causes high structural stress and leads to the loss of electrical contact between active material and the current collector. The Ge@C electrodes, after a short period of fading after the change of specific current, deliver a stable specific capacity for almost 200 cycles with retention values of 90.2, 86.2, and 95.2% for Ge/C-SS750, Ge/C-HT180, and Ge/C-iM750, respectively. This observation demonstrates that the presence of a carbon matrix is useful in stabilizing the electrode by alleviating the stress caused by volume change. Amongst the composites, the electrochemical performance increases in the order of Ge/C-HT180 < Ge/C-SSS750 < Ge/C-iM750. The origin of this improvement is the better chemical contact between Ge and the carbon matrix. The Raman results indicate that this chemical contact is enhanced by the high-temperature treatment, which is in agreement with the improved behavior of Ge/C-SSS750 compared to Ge/C-HT180. In addition, the in situ synthesis induces an improved contact between Ge and the carbon matrix, leading to enhanced electronic and ionic conductivity, which was further analyzed using rate performance and EIS results. Figure 5b shows the rate performance of the Ge@C electrodes at different specific currents. The common phenomenon of capacity reduction at increasing specific currents is observed in all electrodes. However, the higher specific capacity (432.3 mAh·g^−1^) of Ge@C-iM750 indicates an improvement in the kinetics of lithium storage reactions, even under the harshest condition of 5000 mA·g^−1^.

The Nyquist plots of all electrodes (Figure 5c) contain a semicircle in the high-to-medium frequency range and a sloping tail in the low-frequency region. The former component is characteristic of charge transfer resistance, while the latter presents the diffusion of ions in the solid phase. Compared to the pure Ge electrode, the diameters of the semicircle are smaller in the Ge@C electrodes because of the higher electrical conductivity of the additional carbon matrix, indicating the decrease in charge transfer resistance of the composite electrodes. The EIS results were fitted using an equivalent circuit model, including an internal or electrolyte resistance (*R*_e_), a charge transfer resistance (*R*_ct_), and two constant phase elements (CPE) [65]. According to Table 2, the charge transfer resistances of all Ge@C electrodes are much lower than the values of the pure Ge electrode, and Ge/C-iM750 exhibits the lowest value. The ionic conductivity was evaluated using the lithium-ion diffusion coefficient (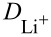) using the following equation:


[2] $$D_{{\text{Li}}^{+}}=\frac{R^{2}T^{2}}{2A^{2}n^{2}F^{4}C^{2}\sigma^{2}},$$

in which, *R* (8.314 J·mol^−1^·K^−1^) is the molar gas constant, *T* (289.15 K) is the temperature, *A* (cm^2^) is the effective surface area of the electrodes, *n* is the number of electrons related to the redox reaction, *F* (96485 C·mol^−1^) is the Faraday constant, *C* (mol·cm^−3^) is the lithium concentration, and σ (Ω·s^−0.5^) is the Warburg coefficient derived from the linear fit of the real part of the impedance (Z′, Ω) as a function of the angular frequency to the power of −0.5 (ω^−0.5^, s^−0.5^):


[3] $$Z=R_{\text{e}}+R_{\text{ct}}+\sigma \cdot \omega^{-0.5}.$$

As shown in Table 2, the 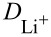 value of Ge/C-iM750 is 2.37 × 10^−16^ cm^2^·s^−1^, 17.7 times higher than that of pure Ge. Therefore, the presence of the carbon matrix, especially in the in situ synthesized composite, plays a key role by not only accommodating the volume variation but also significantly improving the electrical and ionic conductivity of the electrode.

**Table 2:** Impedance parameters obtained from EIS fitting.

Electrode	*R*_e_ (Ω)	*R*_ct_ (Ω)	σ (Ω·s^−0.5^)	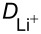 (cm^2^·s^−1^)

Ge	7.3	1023.7	932.1	1.34 × 10^−17^
Ge/C-iM750	10.4	221.4	222.5	2.37 × 10^−16^
Ge/C-HT180	5.2	335.2	646.3	2.79 × 10^−17^
Ge/C-SS750	6.8	267.1	439.8	6.03 × 10^−17^

## Conclusion

In this work, one-pot in situ synthesis of Ge@C composites using magnesiothermic reduction was reported. Structural characterization indicates a good binding between Ge and carbon matrix after the in situ synthesis, which alleviates the structural stress induced by volume change and increases the conduction of electrons and ions in the composite electrodes. These observations are supported by the superior electrochemical performance of the Ge/C-iM750 electrode with a specific capacity of 454.2 mAh·g^−1^ after 200 cycles at a specific current of 1000 mA·g^−1^, which accounted for a retention of 95.2%, and an acceptable rate capacity of 432.3 mAh·g^−1^ at a higher specific current of 5000 mA·g^−1^. Other composites were also prepared using hydrothermal and direct solid-state coupling. However, these routes did not provide such a good chemical contact between the components, as demonstrated by their low specific capacity and poor rate performance.

## Experimental

### Chemicals

The chemicals, including germanium oxide (GeO_2_) (99.99%) from Sigma-Aldrich and hydrochloric acid (HCl) (35–37%), magnesium powder (Mg) (99.95%), ethanol (C_2_H_5_OH) (99.5%), and potassium hydroxide (KOH) (85%) from Xilong, China, were used without further purification. Banana peels were collected from Binh Dinh province, Vietnam.

### Fabrication of activated carbon from banana peel

A procedure similar to our previous work [33] has been used to prepare carbon materials from banana peels. An appropriate amount of banana peels was dried at 80 °C overnight in a vacuum oven and ground into small pieces, then placed in a ceramic crucible and heated to 800 °C for 5 h under argon gas flow with a heating rate of 10 °C·min^−1^. The obtained solid was washed with potassium hydroxide solution (KOH 20%) at 70 °C for 2 h, then leached with 2 M HCl at 70 °C for 15 h and washed several times with de-ionized (DI) water, before being dried in a vacuum oven at 110 °C for 12 h. The product was further heated under air at 300 °C for 3 h. The activated carbon was obtained after washing with 2 M HCL and DI water. It was dried under vacuum and denoted as BC-800.

### In situ fabrication of Ge/C-iM750 composite material

A mixture of germanium dioxide, magnesium, and activated carbon at a mass ratio of 5:4:10, was ground well and transferred into a ceramic crucible. The solid was heated to 750 °C under Ar gas flow for 3 h. The obtained powder was rinsed with 1 M HCl to remove by-products. The sample, obtained after rinsing with DI water and drying at 70 °C for 12 h, was denoted as Ge/C-iM750.

An analogous route to our previous work [29] without adding activated carbon was applied to synthesize pure germanium (denoted as Ge).

### Hydrothermal coupling to synthesize Ge/C-HT180 composite

The hydrothermal synthesis was carried out according to our previous work [33]. Ge and BC-800 at a mass ratio of 2:5 (approximate to that estimated from the Ge/C-750 precursor) were added to a mixture of ethanol and DI water at a ratio of 1:1 (v/v). The dispersion was well stirred in a Teflon beaker, transferred into an autoclave, and held at 180 °C for 12 h. After cooling to room temperature, the solid was collected and rinsed with DI water to neutral pH. The dried solid was denoted as Ge/C-HT180.

### Solid-state reaction coupling to synthesize Ge/C-SS750 composite

Relevant amounts of Ge and BC-800 at a mass ratio of 2:5 were well ground and transferred to a ceramic crucible. The mixture was heated at 750 °C in argon gas flow for 3 h. The obtained solid was re-ground and denoted as Ge/C-SS750.

### Material characterization

X-ray diffraction measurements (XRD, Bruker D8 Advance with Cu Kα radiation (λ = 1.5406 Å) at 40 kV and 40 mA) were carried out for structural and phase information. Infrared (IR, Shimadzu IRAffinity-1S) and Raman (LabRAM HR evolution confocal Raman microscope) spectra were measured for bond analysis. Field-emission scanning electron microscopy (FE-SEM, Hitachi S-4800) and high-resolution transmission electron microscopy (HR-TEM, JEOL JEM-2100F) were conducted for morphology and particle size investigation.

A well-blended mixture of active material, conductive carbon C65, and polyvinylidene fluoride (PVDF) binder with a mass ratio of 75:15:10 in *N*-methyl-2-pyrrolidone solvent was used for fabricating the working electrodes. The obtained slurry was cast onto copper foil using an automatic film coater and dried in a vacuum oven at 80 °C for 10 h. Discs (diameter of 12 mm with a mass loading of the active material of around 1.2 mg·cm^−2^) punched from the film were used as a working electrodes. CR2032 coin cells, in half-cell configuration, were assembled in an argon-filled glovebox (MB 20 G, MBRAUN), with oxygen content and moisture below 1.0 ppm. A disc-shaped lithium metal foil was used as counter electrode, and a glassy carbon fiber pad soaked in 1 M electrolyte of LiPF_6_ in ethylene carbonate/dimethyl carbonate/diethyl carbonate (1:1:1, v/v) with 5% of fluoroethylene carbonate (by mass) was used as separator.

Galvanostatic cycling with potential limitation (GCPL) was performed on a LAND battery testing system CT-2001A in a potential window of 0.1–3.0 V vs Li/Li^+^ at specific currents of 100 mA·g^−1^ for the initial five cycles and 1000 mAh·g^−1^ for the following cycles. The rate performance was measured under the same conditions with different specific currents from 100 to 5000 mA·g^−1^. Cyclic voltammetry (CV) was carried out in the above potential window at a scan rate of 0.1 mV·s^−1^ on a Biologic-MPG2 instrument. Electrochemical impedance spectroscopy (EIS) was carried out at the open-circuit voltage of the assembled cells after 6 h of resting on a Biologic VSP3 potentiostat. A sinusoidal signal with an amplitude of 10.0 mV and a frequency varying exponentially from 10 mHz to 100 kHz was used.
